# The Magnetic Susceptibility Bifurcation in the Ni-Doped Sb_2_Te_3_ Topological Insulator with Antiferromagnetic Order Accompanied by Weak Ferromagnetic Alignment

**DOI:** 10.1186/s11671-021-03637-5

**Published:** 2021-12-20

**Authors:** Shiu-Ming Huang, Pin-Cing Wang, Hao-Lun Jian, Mitch M. C. Chou

**Affiliations:** 1 Department of Physics, National Sun Yat-Sen University, 80424 Kaohsiung, Taiwan; 2 Center of Crystal Research, National Sun Yat-Sen University, 80424 Kaohsiung, Taiwan; 3 Department of Materials and Optoelectronic Science, National Sun Yat-Sen University, 80424 Kaohsiung, Taiwan; 4 Taiwan Consortium of Emergent Crystalline Materials, TCECM, National Sun Yat-Sen University, 80424 Kaohsiung, Taiwan

**Keywords:** Antiferromagnetism, Ferromagnetism topological material, Magnetic susceptibility, Curie–Weiss law

## Abstract

The magnetic susceptibility reveals a discontinuity at Néel temperature and a hysteresis loop with low coercive field was observed below Néel temperature. The magnetic susceptibility of zero field cool and field cool processes coincide at a temperature above the discontinuity, and they split at temperature blow the discontinuity. The magnetic susceptibility splitting is larger at lower external magnetic fields. No more magnetic susceptibility splitting was observed at a magnetic field above 7000 Oe which is consistent with the magnetic anisotropy energy. Our study supports that these magnetic susceptibility characteristics originate from an antiferromagnetic order accompanied by weak ferromagnetism.

## Introduction

Three-dimensional topological insulators possess a linear dispersion gapless surface state that is protected by time-reversal symmetry [1, 2]. The topological surface state consists of spin-filtered Dirac fermions. This spin helical texture of the topological surface state has attracted a great deal of attention due to its possible electric and spin-related applications [3–20]. Aside from the intrinsic exotic characteristics, introduction of magnetization into the topological insulator will modify the electronic. This exchange interaction between conduction electron and magnetic atoms breaks time-reversal symmetry and that opens a gap of Dirac surface state. The Dirac fermion in the surface state becomes massive [1, 2, 21] and leads to many interesting properties, such as quantum anomalous Hall effect, [22, 23] topological magneto-electric effect [24], tunability of chiral edge mode [25, 26] and Majorana braiding [27–29]. The carrier from the topological surface state dominates these magnetoelectrical properties. Many experimental works were performed in Mn, Cr, and V-doped (Bi, Sb)$_{2}$Te$_{3}$ thin films to realize the theoretical prediction [30]. Most of these studies mainly focused on electric-magneto transport properties, such as quantum anomalous Hall effect, topological magnetoelectric effect and related applications. Due to the weak magnetism signal in a thin film with weak magnetic element-doped topological insulator, rare studies on the intrinsic magnetic properties of magnet-doped were reported in magnetic element-doped topological insulators and the related magnetic coupling is not well-explored. To understand the intrinsic novel physical properties of the magnetic element doped topological insulator, especially the role of the magnetic element and the related magnetic interaction coupling, it could be helpful to precisely utilize the magneto properties on the related application.

In this work, we studied the magnetic properties of Ni-doped Sb$_{2}$Te$_{3}$ topological insulator single crystal. A hysteresis loop with a low coercive field was observed below the Néel temperature ($T_{\mathrm {N}}$). The magnetic susceptibility reveals a kick at $T_{\mathrm {N}}$ that is independent of the external magnetic field. The magnetic susceptibility of zero field cool and field cool processes coincide above $T_{\mathrm {N}}$, and they are bifurcation below $T_{\mathrm {N}}$. The magnetic susceptibility splitting is larger at lower external magnetic fields and temperatures. No more magnetic susceptibility splitting is observed at magnetic field above 7000 Oe. Our study supports that these magnetic susceptibility characteristics originates from an antiferromagnetic order accompanied by weak ferromagnetism. The extracted saturated susceptibility goes well with the tendency of the measured magnetic susceptibility cusp. Apart from most reports that the magnetic susceptibility cusp originates from the carrier spin texture at Dirac point of the topological surface state, our results reveal that it might be related to the ferromagnetism of magnetic elements.

## Experimental Method

**Fig. 1:**
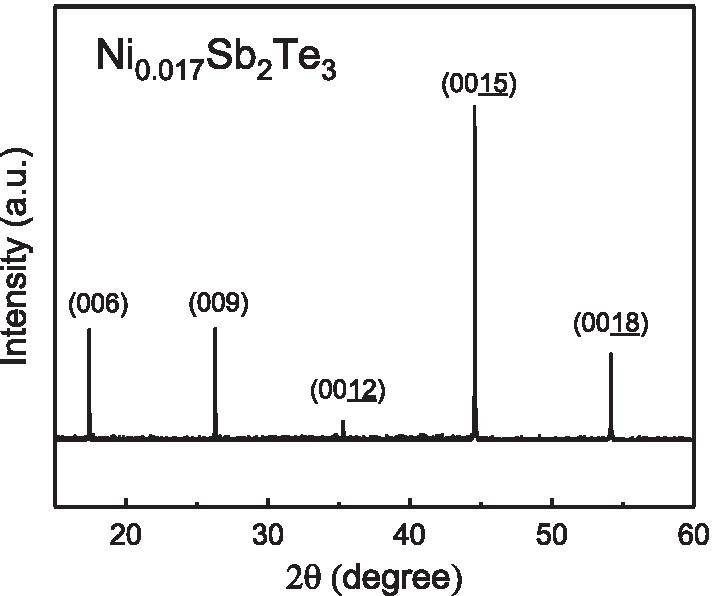
The XRD spectrum of the Ni$_{0.016}$Sb$_{2}$Te$_{3}$ single crystal. It reveals sharp peaks and that supports the highly single-crystallized structure

Single crystals of Sb$_{2}$Te$_{3}$ were grown with a home- made resistance-heated floating zone furnace (RHFZ). The starting raw materials of Sb$_{2}$Te$_{3}$ were mixed according to the stoichiometric ratio. At first, the stoichiometric mixtures of high purity elements Ni (99.995%), Sb (99.995%) and Te (99.995%) were melted at $700 \sim 800 ^{\circ }$C for 20 h and then slowly cooled to room temperature in an evacuated quartz glass tube. The material was used as a feeding rod for the following RHFZ experiment. Our previous work supports that extremely high crystal uniformity in topological insulator crystals can be obtained through the RHFZ method. After growth, the crystals were then furnace cooled to room temperature. The as-grown crystals were cleaved along the basal plane, with a silvery shiny mirror-like surface, and then prepared for further experiments. The Energy-dispersive spectrum (EDS) results support that the $\mathrm {Ni} : \mathrm {Sb} : \mathrm {Te} = 0.017 : 2 : 3$. Figure 1 shows the X-ray Diffraction (XRD) spectrum. It reveals sharp peaks and these peaks are consistent with the database of Sb$_{2}$Te$_{3}$. This confirms that our sample is highly crystallized. The Ni atoms are expected to be uniformly and randomly distributed in the single crystal. The crystal size is 3-mm long, 2-mm wide and 0.42-mm thickness. Magnetism measurements were performed using the standard technique in a commercial apparatus (Quantum Design MPMS) with a magnetic field of up to 7 T. The magnetic field was applied perpendicular to the large cleaved surface.

## Results and Discussion

**Fig. 2:**
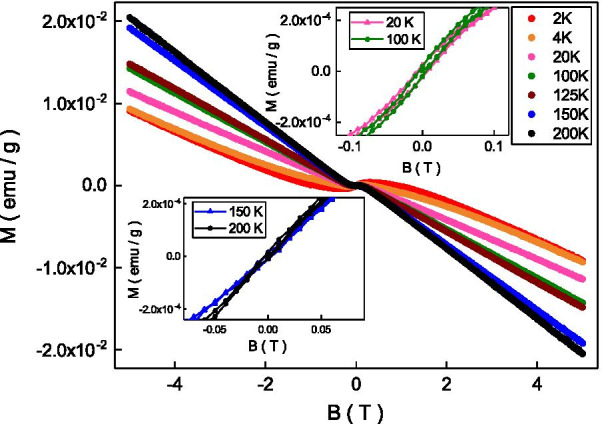
The susceptibility as a function of magnetic fields from 2 to 200 K. It reveals the diamagnetism at high magnetic fields. Top-right inset: The hysteresis loop was observed at temperature below 125 K. Bottom-left inset: No hysteresis loop was observed at temperature above 125 K

Figure 2 shows the magnetization as a function of magnetic fields at different temperatures, and it revealed the diamagnetic characteristic at a wide range of magnetic fields and temperatures. This diamagnetism comes from the carrier spin and it is consistent with the previous reports in BSTS topological insulators [31]. As shown in the top-right inset, different from previous reports, a hysteresis loop was observed at temperatures below 125 K. The coercive field of the hysteresis loop shows weak temperature dependence and it is roughly 50 Oe. The remanent and saturated magnetization of the hysteresis loop is about $10^{-5}$ emu/g and $10^{-4}$ emu/g at 100 K. The low coercive field, the small remanent, and the small saturated magnetization indicate weak ferromagnetism. As shown in the bottom-left inset, no clear hysteresis loops were observed at temperatures above 125 K. The ferromagnetism originates from the aligned magnetic moments of the magnetic elements. The thermal energy might randomize the aligned magnetic moment and smear out the ferromagnetism above a critical temperature. Our observation indicates that the system reveals a weak ferromagnetism transition around 120 K.

**Fig. 3:**
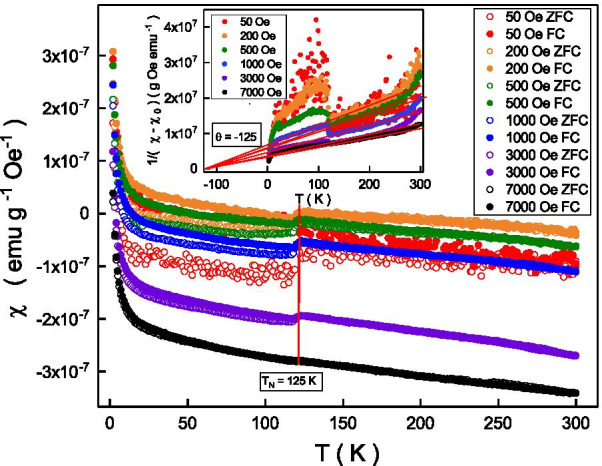
The magnetic susceptibility of field cooled and zero-field cooled processes at different external magnetic fields. The magnetic susceptibility of field cooled and zero-field cooled coincides above 125 K and bifurcates below 125 K. A larger magnetic susceptibility splitting at lower external magnetic fields and temperatures. No more magnetic susceptibility splitting is observed at magnetic field above 7000 Oe. Top-right inset: The magnetic susceptibility follows the Curie-Weiss law

**Fig. 4:**
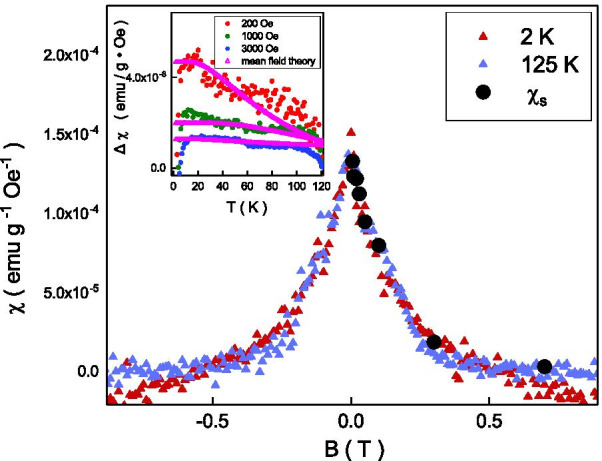
Top-left inset: The magnetic susceptibility difference of field cooled and zero-field cooled follows the mean field theory. The extracted saturated susceptibility goes well with the tendency of the measured magnetic susceptibility cusp

To investigate the intrinsic magnetism characteristic of the observed weak ferromagnetic transition, the temperature-dependent magnetic susceptibility was performed through field-cooled and zero-field-cooled processes. Figure 3 shows the magnetic susceptibility of field-cooled and zero-field cooled processes at different external magnetic fields. The magnetic susceptibility increases as temperature decreases. It reveals a discontinuity at 125 K ($T_{\mathrm {N}}$) and the $T_{\mathrm {N}}$ is independent of the external magnetic fields. The $T_{\mathrm {N}}$ is the Néel temperature and the detailed mechanism will be discussed and clarified below. The magnetic susceptibility of field-cooled and zero-field-cooled coincides above $T_{\mathrm {N}}$ and bifurcates below $T_{\mathrm {N}}$. A larger magnetic susceptibility splitting is observed at lower external magnetic fields. Our experimental result shows that this discontinuity and the magnetic susceptibility splitting is no more observed at magnetic field higher than 7000 Oe. It is worthy to notice that the signal fluctuation at the magnetic field of 50 Oe is obviously larger than other magnetic fields. One of the possible reasons is that the magnetic moment alignment is metastable at the 50 Oe that is close to the hysteresis loop coercive field. As shown in Fig. 2, the hysteresis loop was only observed below 125 K that is the same as the critical temperature of the magnetic susceptibility bifurcation in Fig. 3. This indicates the observed magnetic susceptibility splitting might be related to the weak ferromagnetic below the $T_{\mathrm {N}}$. It is known that the ferromagnetic effect would be smeared out by thermal energy and the magnetic susceptibility above the critical temperature could be described by the Curie-Weiss law, $\chi = \chi _{0} + \frac{C}{T-\theta }$, where $\chi$ is the measured magnetic susceptibility, $\chi _{0}$ is the magnetic susceptibility at 0 K, *C* is the Curie constant that is corresponding to the Bohr magneton, *T* is the temperature, and $\theta$ is the Curie temperature [32]. The inset of Fig. 4 shows the temperature dependence of zero-field cooled $\frac{1}{\chi - \chi _{0}}$ at different external magnetic fields. The $\frac{1}{\chi -\chi _{0}}$ is proportional to a temperature between 125 and 250 K, and the slope is larger at lower external magnetic fields. The slope is related to the Curie constant. The linear extrapolation of the $\frac{1}{\chi -\chi _{0}}$ between 125 and 250 K of all external magnetic fields coincide at -125 K. Following the Curie-Weiss law, this value is corresponding to the $\theta$. The negative $\theta$ (-125 K) indicates that it is an antiferromagnetic system below the $T_{\mathrm {N}}$ and $T_{\mathrm {N}}$ is known as Néel temperature [33]. The absolute value of the $\theta$ is consistent with the observed $T_{\mathrm {N}}$ in Fig. 3 , and the critical temperature to observe the hysteresis loop (125 K) in Fig. 2. These observations indicate that weak ferromagnetism and antiferromagnetism coexist below $T_{\mathrm {N}}$.

As shown in the inset of Fig. 3, the Curie constant, *C*, is larger at higher magnetic fields. Following the Langevin paramagnetic function, *C* could be expressed as $C=\frac{N\mu _{0}\mu ^{2}}{3k_{\mathrm {B}}T}$ where *N* is the number of magnetic elements per unit gram, $\mu$ is the effective moment of a magnetic element, $\mu _{0}$ is the vacuum permeability and $k_{\mathrm {B}}$ is the Boltzmann constant [34]. The estimated $\mu$ at 200 Oe is about 3.5 $\mu _{\mathrm {B}}$ that is closed to the theoretical value of 3.32 $\mu _{\mathrm {B}}$ [35]. This confirms that magnetism behavior could be explained by the Curie-Weiss law.

The magnetic moment is randomly frozen in the zero-field-cool and frozen along the external magnetic field direction in the field-cool. The magnetic susceptibility bifurcation originates from the magnetic anisotropy. This feature might be a characteristic for an antiferromagnetism order accompanied by weak ferromagnetism; ferromagnetic moments of domains freeze in a random direction in zero-field-cool, while they are forced to align along the applied magnetic field upon cooling across $T_{\mathrm {N}}$ in field cool [36]. As discussed above, it composes of both weak ferromagnetic and antiferromagnetic characteristics below $T_{\mathrm {N}}$ in our system. The weak ferromagnetic alignment would slightly break the antiferromagnetism order and induce the magnetic anisotropy. The magnetic susceptibility bifurcation could be understood as weak ferromagnetism in an antiferromagnetic system. These results support the observed magnetic susceptibility bifurcation below 125 K is the magnetic characteristic of the weak ferromagnetism in an antiferromagnetic system. The different susceptibility splitting at different external magnetic field might originate from the different partial polarization level of antiferromagnetism at external magnetic fields.

Following the mean field theory, [37] the $T_{\mathrm {N}}$ is related to the exchange coupling strength, $J_{0}$, and it could be expressed as $T_{\mathrm {N}}=\frac{S(S+1)}{3k_{\mathrm {B}}T}J_{0}$, where *S* is the spin moment, $k_{\mathrm {B}}$ is Boltzmann constant. The $J_{0}$ would go to $4.28 \times 10^{22}$ joule in our system with $T_{\mathrm {N}}$ = 125 K. The mean field theory supports that the magnetization is related to the thermal energy by a factor of $e^{\frac{-J_{0}S}{k_{\mathrm {B}}T}}$. The magnetic susceptibility could be expressed as $\chi = \chi _{\mathrm {S}}(1-e^{\frac{-J_{0}S}{k_{\mathrm {B}}T}})$, where $\chi _{\mathrm {S}}$ is the saturated magnetic susceptibility. The magnetic susceptibility splitting, $\chi _{\mathrm {FC}}-\chi _{\mathrm {ZFC}}$ could be expressed as $\chi _{\mathrm {S}}e^{\frac{-J_{0}S}{k_{\mathrm {B}}T}}$. The $\chi _{\mathrm {S}}$ is sensitive to external magnetic fields. As shown in the inset of Fig. 4, this equation could explain our experimental result well at a wide range of temperatures and external magnetic fields. The extracted $\chi _{\mathrm {S}}$ is a function of external magnetic fields. To further examine the result, the magnetic field dependent susceptibility is performed at temperatures below $T_{\mathrm {N}}$, and it shows a cusp at zero magnetic fields. This magnetic susceptibility cusp at zero magnetic field is widely observed in topological materials, and it is speculated to originate from the free-aligned spin texture at the Dirac point [38]. The Angle-resolved photoemission spectroscopy (ARPES) reveals that the Fermi level lies below the Dirac point in our Sb$_{2}$Te$_{3}$ [39]. The observed cusp should not originate from the spin texture at the Dirac point. On the other hand, the coercive field of the hysteresis loop is about 50 Oe that is two orders of magnitude lower than full width at half maximum of the cusp, 0.4 T, and the hysteresis loop should not be the main source of the observed cusp. As shown in the inset of Fig. 4, the extracted magnetic field-dependent $\chi _{\mathrm {S}}$ follows the same magnetic field tendency of the measured magnetic susceptibility. This indicates that the widely observed susceptibility cusp might originate from the antiferromagnetic order accompanied by weak ferromagnetism alignment.

**Fig. 5:**
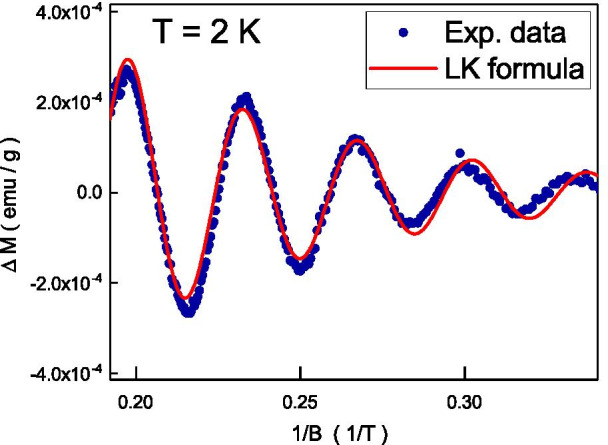
The dHvA oscillations as a function of inverse magnetic fields. The experimental result fits well with the theoretical equation

Following the analysis, the susceptibility bifurcation originates from the magnetism of weak ferromagnetism order accompanied by antiferromagnetism. The magnetic susceptibility splitting is related to the magnetocrystalline anisotropy. Herewith, we further estimate the magnetocrystalline anisotropy energy, $\Delta E = \frac{M_{\mathrm {S}}H_{\mathrm {C}}V}{2}$, where $H_{\mathrm {C}}= 50$ Oe, $M_{\mathrm {S}}= 1.81\times 10^{-11}$ J/T and $V=2.5\times 10^{-9}$ m$^{3}$ in our system, and the $\Delta E \sim 1.13 \times 10^{22}$ Joule [40]. Following the magnetic moment energy, $g\mu _{\mathrm {B}}B$, one could estimate that the magneto crystalline anisotropy energy will be lower than the magnetic moment energy at $B > 0.61$ T. That is consistent with our observation that the magnetic susceptibility splitting is no longer observed at external magnetic fields above 0.7 T.

Figure 5 shows the magnetic susceptibility as a function of 1/*B* and it shows periodic oscillations. This is known as the De Haas-Van Alphen effect (dHvA) oscillations that originate from the orbital motion of itinerant electron at high magnetic fields [41]. We analyze the dHvA oscillations by fitting the oscillatory magnetization to the Lifshitz-Kosevich (LK) formula [42], $\Delta M \propto -R \sin [2\pi (\frac{F}{B}-\delta _{p})]$. *R* is related to the carrier scattering rate, Zeeman effect, and Landau level broadening [43]. The oscillation is described by a sinusoidal term that contains the phase factor $\delta _{p}$. $\delta _{p}$ is related to the Berry phase ($\Phi _{B}$), $\delta _{p} = \frac{1}{2}-\frac{\Phi _{B}}{2\pi }$. The dimension of the Fermi pocket characterizes the value $\delta _{p}$. As shown in Fig. 5, the theoretical equation fits well with our experimental result and the extracted $\delta _{p}=0.43$ and $F = 29.8$ T. That is consistent with the theoretical prediction and the observed dHvA comes from the topological surface state. Following the Onsager relation [44], $F=\frac{\hbar K_F^{2}}{2\pi }$, one could estimate that $K_{F} = 0.030$Å^−^^1^  is consistent with the reported value from ARPES. These results suggest that the dHvA oscillations originate from the topological surface state.

## Conclusion

In this work, we studied the magnetic behavior of Ni-doped Sb$_{2}$Te$_{3}$ topological insulator single crystal. A hysteresis loop with low a coercive field was observed below the Néel temperature. The magnetic susceptibility reveals a kick at Nèel temperature that is independent of the external magnetic field. The magnetic susceptibility of zero field cool and field cool processes are coinciding above the Néel temperature, and they are bifurcation below Néel temperature. The magnetic susceptibility splitting is larger at a lower external magnetic field. No more magnetic susceptibility splitting is observed when the magnetic moment anisotropy energy is lower than the magnetic moment energy at 0.7 T. Our study supports that these magnetic susceptibility characteristics originate from an antiferromagnetic order accompanied by weak ferromagnetism. The extracted saturated magnetic susceptibility goes well with the tendency of the measured magnetic susceptibility cusp. This indicates that the widely observed susceptibility cusp might originate from the weak ferromagnetism. The dHvA oscillation is consistent with the theoretical prediction. This supports that observed dHvA oscillation comes from the topological surface state.

## Data Availability

The datasets generated during and/or analyzed during the current study are available from the corresponding authors on reasonable request.

## Abbreviations

XPD: X-ray diffraction
EDS: Energy-dispersive X-ray spectroscopy
ARPES: Angle resolved photoemission spectroscopy
dHvA: De Haas-Van Alphen
